# Oppositional Regulation of Noxa by JNK1 and JNK2 during Apoptosis Induced by Proteasomal Inhibitors

**DOI:** 10.1371/journal.pone.0061438

**Published:** 2013-04-11

**Authors:** Sabine Pietkiewicz, Dennis Sohn, Roland P. Piekorz, Susanne Grether-Beck, Wilfried Budach, Kanaga Sabapathy, Reiner U. Jänicke

**Affiliations:** 1 Laboratory of Molecular Radiooncology, Clinic and Policlinic for Radiation Therapy and Radiooncology, University of Düsseldorf, Düsseldorf, Germany; 2 Institute for Biochemistry and Molecular Biology II, University of Düsseldorf, Düsseldorf, Germany; 3 IUF-Leibniz Research Institute for Environmental Medicine, Düsseldorf, Germany; 4 Division of Cellular & Molecular Research, Humphrey Oei Institute of Cancer Research, National Cancer Centre, Singapore, Singapore; German Cancer Research Center, Germany

## Abstract

Proteasome inhibitors (PIs) potently induce apoptosis in a variety of tumor cells, but the underlying mechanisms are not fully elucidated. Comparing PI-induced apoptosis susceptibilities of various mouse embryonic fibroblast (MEF) lines differing in their c-jun N-terminal kinase (JNK) 1 and 2 status, we show that several hallmarks of apoptosis were most rapidly detectable in JNK2−/− cells, whereas they appeared only delayed and severely reduced in their intensities in cells expressing JNK2. Consistent with our finding that PI-induced apoptosis requires de novo protein synthesis, the proteasomal inhibitor MG-132 induced expression of the BH3-only protein Noxa at the transcriptional level in a JNK1-dependent, but JNK2-opposing manner. As the knockdown of Noxa blocked only the rapid PI-induced apoptosis of JNK2−/− cells, but not the delayed death occurring in JNK1−/− and JNK1+/+ cells, our data uncover a novel PI-induced apoptosis pathway that is regulated by the JNK1/2-dependent expression of Noxa. Furthermore, several transcription factors known to modulate Noxa expression including ATF3, ATF4, c-Jun, c-Myc, HIF1α, and p53 were found upregulated following MG-132 exposure. From those, only knockdown of c-Myc rescued JNK2−/− cells from PI-induced apoptosis, however, without affecting expression of Noxa. Together, our data not only show that a rapid execution of PI-induced apoptosis requires JNK1 for upregulation of Noxa via an as yet unknown transcription factor, but also that JNK2 controls this event in an oppositional manner.

## Introduction

In a plethora of in vitro studies it has been extensively demonstrated that inhibition of the proteasome for instance by the tripeptide aldehyd MG-132 or the dipeptide boronate bortezomib (Velcade^™^) selectively kills tumor cells of varying origin (reviewed in Ref. [1]). Proteasomal inhibitors (PIs) also sensitize cells to radio- and chemotherapy and even to apoptosis induced by death receptor ligands [2], [3]. However, as the proteasome targets not only pro-, but also anti-apoptotic proteins, a successful combination therapy requires a successive application of first the apoptosis-inducing agent ensuring the breakdown of anti-apoptotic proteins followed by the PI treatment that then prevents degradation of the generated pro-apoptotic proteins [4]. Nevertheless, bortezomib was the first PI used in clinical trials and approved to treat patients suffering from multiple myeloma or mantle cell lymphoma [5].

Although the new generation of proteasome inhibitors such as salinosporamide and carfilzomib appear to exhibit somewhat different mechanisms of action than bortezomib, central to apoptosis induction by many PIs is certainly the mitochondrial or intrinsic death pathway, as their cytotoxic activity is almost completely abrogated in cells deficient for Bax and Bak [6]. Consistently, a number of studies strongly implicated certain pro-apoptotic BH3-only proteins in PI-induced apoptosis [7]. For instance, the pro-apoptotic cleavage product of Bid, t-Bid, is degraded by the proteasome and treatment of HeLa cells with MG-132 resulted in accumulation of t-Bid and sensitized the cells to death receptor-induced apoptosis [8]. Also Bik and Bim were found to be upregulated following PI treatment and cells deficient for both or cells in which Bik and Bim were down regulated by RNA interference were refractory to its cytotoxic action [9], [10]. Likewise, different PIs including bortezomib and MG-132 were shown to induce expression of Noxa in several tumor models both at the protein and mRNA level and siRNA-mediated knockdown of Noxa partially rescued various tumor cells from PI-induced apoptosis [11]–[13]. Expression of other Bcl-2 family members such as Puma, Bax, Bak, Bcl-2, and Bcl-X_L_ remained mostly unaffected following treatment of different cell lines with PIs.

Several signaling pathways have been shown to play a role in PI-induced cytotoxicity including stabilization of the tumor suppressor protein p53, inhibition of the nuclear factor-κB (NF-κB), or induction of an ER-stress response [14]–[16]. As Noxa was first identified as a p53 target gene [17], the stabilization and activation of p53 would have been an attractive possibility for apoptosis induction by PIs. However, PI-mediated tumor cell killing was also observed in p53-deficient cells and independently of NF-κB inhibition suggesting that other signaling pathways targeted by the proteasome are even more crucial for cell death induction by PIs [15], [18]. One of those might be instigated by members of the mitogen-activated protein (MAP) kinase family, the c-Jun N-terminal kinases (JNKs) that were reproducibly found to be activated in PI-treated cells [19], [20]. More intriguingly, inhibition of JNK activity by either dominant-negative JNKs or by RNA interference rendered the cells resistant toward cell death induction by PIs [20], [21]. Thus, it appears that JNKs, in addition to several other pathways in which they were shown to contribute to apoptosis signaling [22], are also crucial players in PI-induced apoptosis.

Three JNK isoforms (JNK1, 2 and 3) with different splice variants are expressed either ubiquitously (JNK1 and JNK2) or preferentially in neuronal and heart tissues (JNK3) [23]. They were originally identified by their ability to specifically phosphorylate and activate c-Jun, a constituent of the activator protein-1 (AP-1) transcription factor that is involved in the increased expression of several pro-apoptotic genes such as TNF-α, Fas-ligand, Bak and Bim [22]. Although silencing of the c-Jun/AP-1 pathway conferred resistance to JNK-mediated apoptosis in several cellular systems, the observed stimulus- and cell type-dependent manner of protection suggests participation of other downstream effectors [24]. Indeed, JNKs appear to control apoptosis in quite a versatile manner as they not only phosphorylate and activate other pro-apoptotic transcriptions factors including p53 and c-Myc, but also several Bcl-2 family proteins causing inhibition of pro-survival members such as Bcl-2, Bcl-X_L_ and Mcl-1 and activation of pro-apoptotic members such as Bim and Bad [22]. However, although these phosphorylation events are consistent with the observation that JNKs are required for stress-induced activation of the mitochondrial death pathway [25], their contributions to apoptosis are controversially discussed [23]. In addition, it is unknown whether JNK1 and JNK2 exhibit redundant or specific functions in PI-induced apoptosis and whether they are involved in the induction of Noxa.

To elucidate these questions in more detail, we compared PI-induced apoptosis signaling of immortalized mouse embryonic fibroblasts (MEF) that differ in their JNK1 and/or JNK2 status. In addition to our findings that JNK1 greatly accelerates de novo expression of Noxa and subsequent apoptosis, we also observed that both processes were strongly impaired in the presence of JNK2 implying oppositional roles for these isoforms in PI-induced apoptosis. Intriguingly, although either knockdown of the transcription factor c-myc or Noxa protected JNK2−/− cells from PI-induced apoptosis, they were found to function independently of each other.

## Results

### Proteasomal inhibitors require JNK1 and de novo protein synthesis to rapidly induce apoptosis

**Figure 1:**
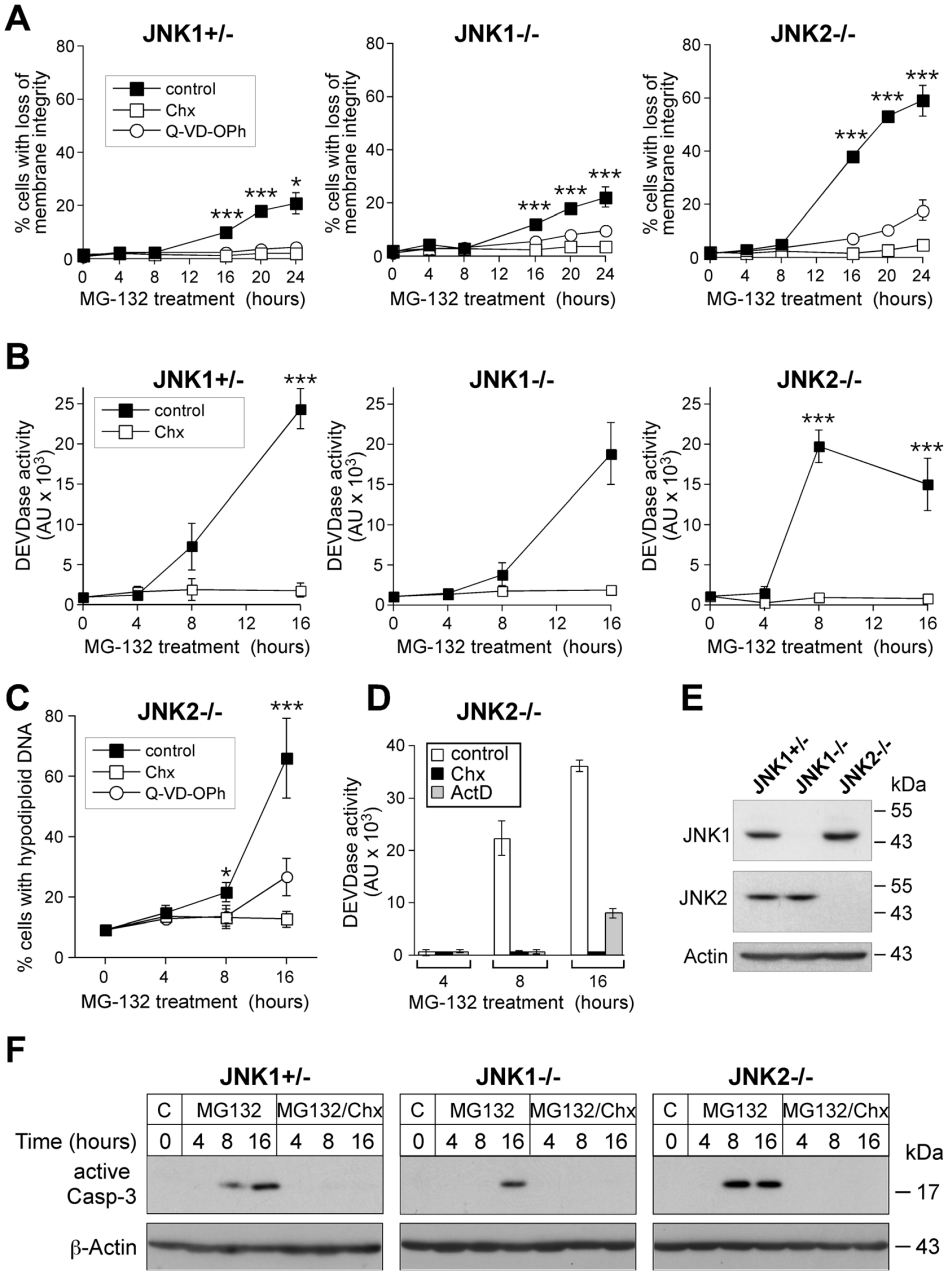
Rapid induction of apoptosis by MG-132 only in JNK2−/− cells. The three MEF lines were exposed to MG-132 for the indicated times in the absence or presence of Chx, ActD or Q-VD-OPh. Cell death was determined either cytometrically by quantification of propidium iodide-stained cells (**A**) and hypodiploid nuclei (**C**), or by fluorometric assessment of caspase-3-like DEVDase activities (**B**, **D**). Values represent the mean of three (**A–C**) and two (**D**) independent experiments +/− SD. (**E**) Extracts of the indicated MEF lines were analyzed by Western blotting for their JNK status using the detection of β-Actin as loading control. (**F**) Western blots showing the status of active caspase-3 and β-actin in extracts of the three MEF lines treated as indicated. One representative experiment out of three is shown. For all panels, * p<0.05; *** p<0.005 according to ANOVA Tukeýs test in a time-matched comparison of cells that were treated with MG-132 alone vs. cells treated with MG-132 in the presence of Chx or Q-VD-OPh, respectively.

We first compared JNK2-deficient MEFs with MEFs that are either heterozygously or homozygously deleted for JNK1 (Fig. 1E) [26] with regard to their sensitivities toward cell death induction by the proteasomal inhibitor MG-132. Whereas JNK2-deficient MEFs were efficiently and rapidly killed by MG-132 in a time- and dose-dependent manner, JNK1+/− and JNK1−/− cells showed a significantly delayed and only moderate sensitivity toward this treatment (Figs. 1A, S1A; data not shown). Cell death was almost completely blocked in the presence of the pan-caspase inhibitory peptide Q-VD-OPh, indicating that MG-132 induces apoptosis in these cells (Fig. 1A). Further FACS analyses confirmed this mode of cell death, as MG-132-treated JNK2-deficient cells displayed extensive DNA fragmentation as evidenced by the time-dependent increase of the apoptotic sub-G1 peak and its susceptibility to caspase inhibition (Fig. 1C). Accordingly, processing and activity of caspase-3 peaked in JNK2-deficient cells after an 8 hour MG-132 treatment, whereas these events were completely undetectable or became only barely visible at this time point in similar treated JNK1−/− and JNK1+/− MEFs, respectively (Figs. 1B, 1F, S1B). Although caspase-3 activities significantly increased when the latter two cell lines were exposed to MG-132 for 16 hours (Figs. 1B, 1F), our data suggest that JNK1 is required for a rapid apoptosis induction by MG-132. Furthermore, MG-132-induced cell death and caspase-3 activation were completely abrogated in the presence of actinomycin D (ActD) (Fig. 1D) or cycloheximide (Chx) (Fig. 1), suggesting the requirement for *de novo* protein synthesis in these JNK1-dependent processes.

For specificity reasons we also analyzed cell death induction by other proteasomal inhibitors such as ALLN and ALLM that are effective and weak proteasomal inhibitors, respectively, or clasto-lactacystin β-lactone (CLC) and bortezomib that are among the most selective PIs known. Except for the weak inhibitor ALLM, all the compounds tested reproducibly induced apoptosis in a dose-dependent manner almost exclusively in JNK2−/− cells, whereas these treatments triggered a significantly weaker response in JNK1+/− and JNK1−/− cells. (Fig. S1C). These results strongly indicate that the observed apoptosis induction by these compounds is indeed caused by their ability to block the 26S proteasome. In addition, and similar to our results obtained with MG-132, apoptosis induction by these PIs was completely abrogated in the presence of Chx (Fig. S2). Thus, consistent with the fact that JNKs phosphorylate and activate numerous transcription factors involved in apoptosis signaling, our results demonstrate the absolute necessity of *de novo* protein synthesis in the JNK1-dependent apoptosis pathway induced by PIs.

### MG-132 induces a JNK1-dependent upregulation of Noxa at the transcriptional level

**Figure 2:**
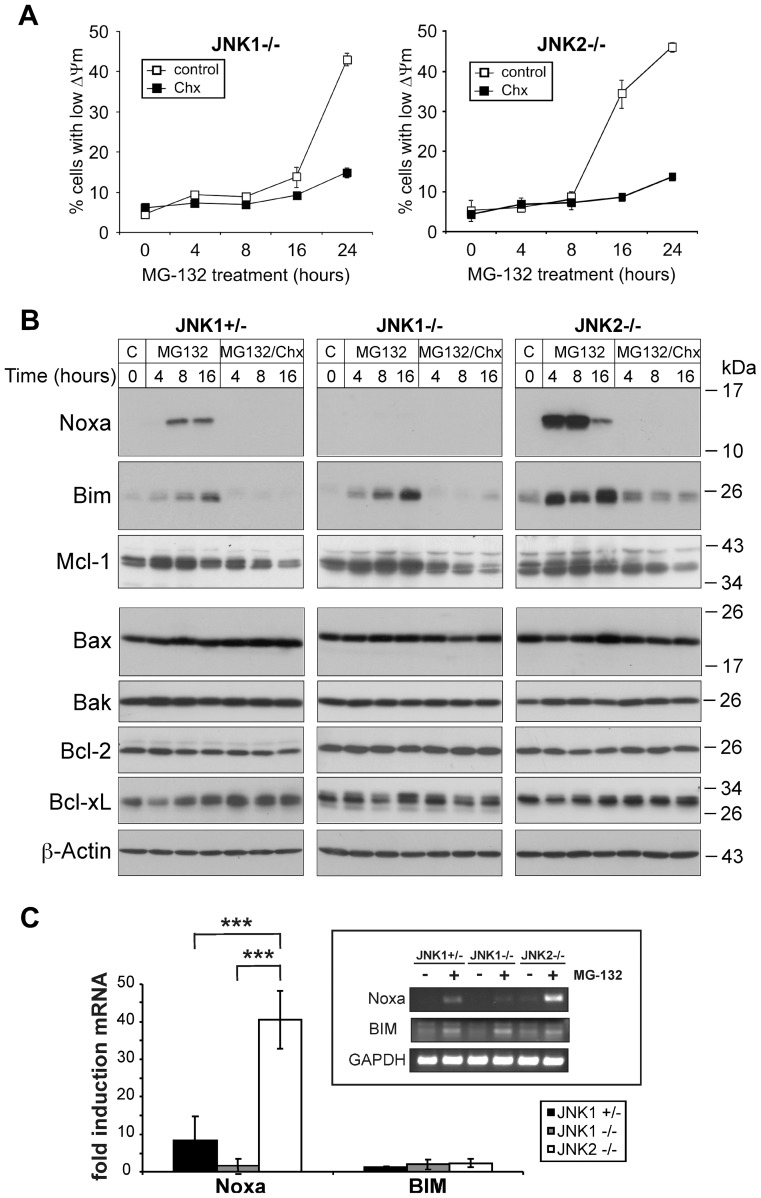
JNK1-dependent upregulation of Noxa mRNA and protein by MG-132. (**A**) Measurement of the mitochondrial membrane potential (ΔΨm) of JNK1−/− and JNK2−/− cells exposed to MG-132 in the absence (control) or presence of Chx. Values represent the mean of two independent experiments +/− SD. (**B**) Western blots showing the status of the indicated Bcl-2 family members and β-actin in extracts of the three MEF lines treated as indicated. One representative experiment out of three is shown. (**C**) Semi-quantitative PCR for determination of MG-132-induced expression of Noxa and Bim mRNAs in the indicated MEF lines normalized to GAPDH mRNA. Data are the mean of three independent experiments +/− SD. *** p<0.005 according to ANOVA Tukeýs test as indicated. The inlet shows one representative agarose gel.

Central to apoptosis induction by PIs and also JNKs is certainly the mitochondrial or intrinsic death pathway, as both are known to modulate expression and activity of several components involved in this pathway including transcription factors and members of the Bcl-2 protein family [6], [22]. Therefore, we analysed the mitochondrial membrane potential (ΔΨ_m_) of the individual MEF lines treated with MG-132. In agreement with our apoptosis data (Figs. 1, S1, S2), we found indeed that MG-132 induced a transcription/translation-dependent loss of ΔΨ_m_ that was much earlier evident in JNK2-deficient cells than in JNK1−/− cells (Fig. 2A).

We also assessed the influence of MG-132 on the expression of various members of the Bcl-2 protein family in these MEF lines. Whereas steady state levels of the pro-apoptotic multi domain proteins Bax and Bak as well as the anti-apoptotic Bcl-2 and Bcl-xL proteins were not altered by MG-132 in any of the MEF lines, Mcl-1 levels expectedly increased upon this treatment in accordance with the susceptibility of this protein to proteasomal degradation (Fig. 2B). Although this increase was prevented in the presence of Chx, it occurred in all cell lines to a comparable extent and thus in a JNK-independent manner. More interestingly, significant and JNK-dependent changes were observed with regard to expression of the BH3-only proteins Bim and Noxa (Fig. 2B). Both pro-apoptotic proteins were nearly undetectable in untreated cells, but were readily induced by MG-132 particularly in JNK2−/− cells that were most sensitive to PIs. In these cells, Bim and Noxa induction following MG-132 exposure was not only more pronounced, but proceeded also with much faster kinetics than in similar treated JNK1+/− and JNK1−/− cells. In addition, MG-132-induced expression of both proteins was almost completely abolished in the presence of cycloheximide, an observation consistent with the thereby caused apoptosis resistance of JNK2−/− cells. Despite this, however, it appears that only Noxa fulfills the criteria of a PI-induced JNK1-dependent apoptosis mediator in an unconfined manner, as this BH3-only protein was most abundantly induced by MG-132 in JNK2−/− cells, but completely undetectable or only weakly expressed in similar treated JNK1−/− and JNK1+/− cells, respectively. Bim on the other hand was induced by MG-132 in a JNK1-independent manner, albeit most pronounced in JNK2−/− cells, and was, in contrast to Noxa, still detectable following cycloheximide treatment.

Consistent with these findings and the observed dependency of PI-induced apoptosis on *de novo* protein synthesis, we found that MG-132 exerts a profound effect only on the transcriptional induction of Noxa mRNA, but not on Bim mRNA (Fig. 2C). Similar to the JNK1-dependent increase in Noxa protein (Fig. 2B), MG-132 induced a dramatic (approximately 40-fold) upregulation of Noxa mRNA in JNK2−/− cells (Fig. 2C). In JNK1+/− cells in contrast, MG-132 increased Noxa mRNA expression only by approximately 8-fold and completely failed to do so in the complete absence of JNK1 in JNK1−/− cells. In contrast, expression of Bim mRNA was only slightly affected by MG-132 in either cell line, suggesting that Noxa, but not Bim, is responsible for the observed PI-induced and JNK1-dependent execution of apoptosis.

### JNK1-dependent upregulation of Noxa is required for PI-induced apoptosis

**Figure 3:**
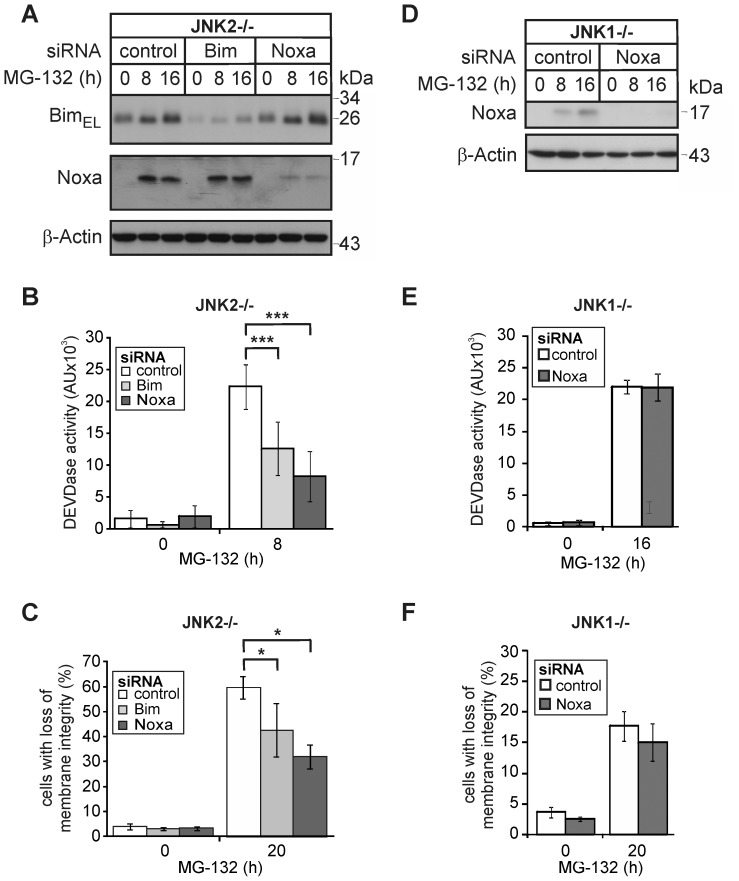
Knockdown of Noxa protects JNK2−/− cells, but not JNK1−/− cells from MG-132-induced apoptosis. (**A**, **D**) Western blots showing the status of BimEL, Noxa and β-actin in JNK2−/− and JNK1−/− cells that were either left untreated or exposed for the indicated times to MG-132 72 hours post transfection with control, Noxa or Bim siRNAs. One representative experiment out of five and three is shown, respectively. Please note that the autoradiograph in **D** was overexposed in order to detect trace amounts of Noxa in JNK1−/− cells. (**B**, **E**) Fluorometric determination of caspase-3 (DEVDase) activities in JNK2−/− and JNK1−/− cells treated as described in **A** and **D**, respectively. Please note that DEVDase activities of JNK2−/− and JNK1−/− cells were analyzed at different time points (8 h and 16 h) according to their sensitivities. (**C**, **F**) Cytometric determination of cell death (propidium iodide uptake) in JNK2−/− and JNK1−/− cells treated as described in **A** and **D**, respectively. Values are the mean of four and three independent experiments, respectively, +/− SD. * p<0.05; *** p<0.001 according to ANOVA Tukeýs test (**B**) or ANOVA on ranks Student-Newman-Keuls-Method (**C**) in an all pairwise multiple comparison as indicated.

To test the hypothesis that the JNK1-dependent transcriptional upregulation of Noxa represents a key event in PI-induced apoptosis, we analyzed the consequences of a siRNA-mediated knockdown of Noxa or Bim. Although both siRNAs caused a comparable reduction in their respective protein targets (Fig. 3A), the knockdown of Noxa was reproducibly more effective in preventing MG-132-induced caspase-3 activation and apoptosis than depletion of Bim (Figs. 3B, 3C). Whereas the Noxa siRNA reduced these events by approximately 64% and 50%, only 41% and 30% inhibition of caspase-3 activation and apoptosis were observed following knockdown of Bim, respectively. Thus, while Bim surely participates in PI-induced apoptosis signaling, JNK1-induced Noxa appears to be the more crucial event. In support of this idea, we found that knockdown of residual Noxa had even after 16 h no effect on MG-132-induced caspase-3 activation and apoptosis in JNK1−/− cells (Fig. 3D**–**F), which is consistent with the delayed JNK1-independent death pathway induced by PIs in these cells (Fig. 1).

### JNK1 and JNK2 oppositional regulate PI-induced Noxa expression and apoptosis

**Figure 4:**
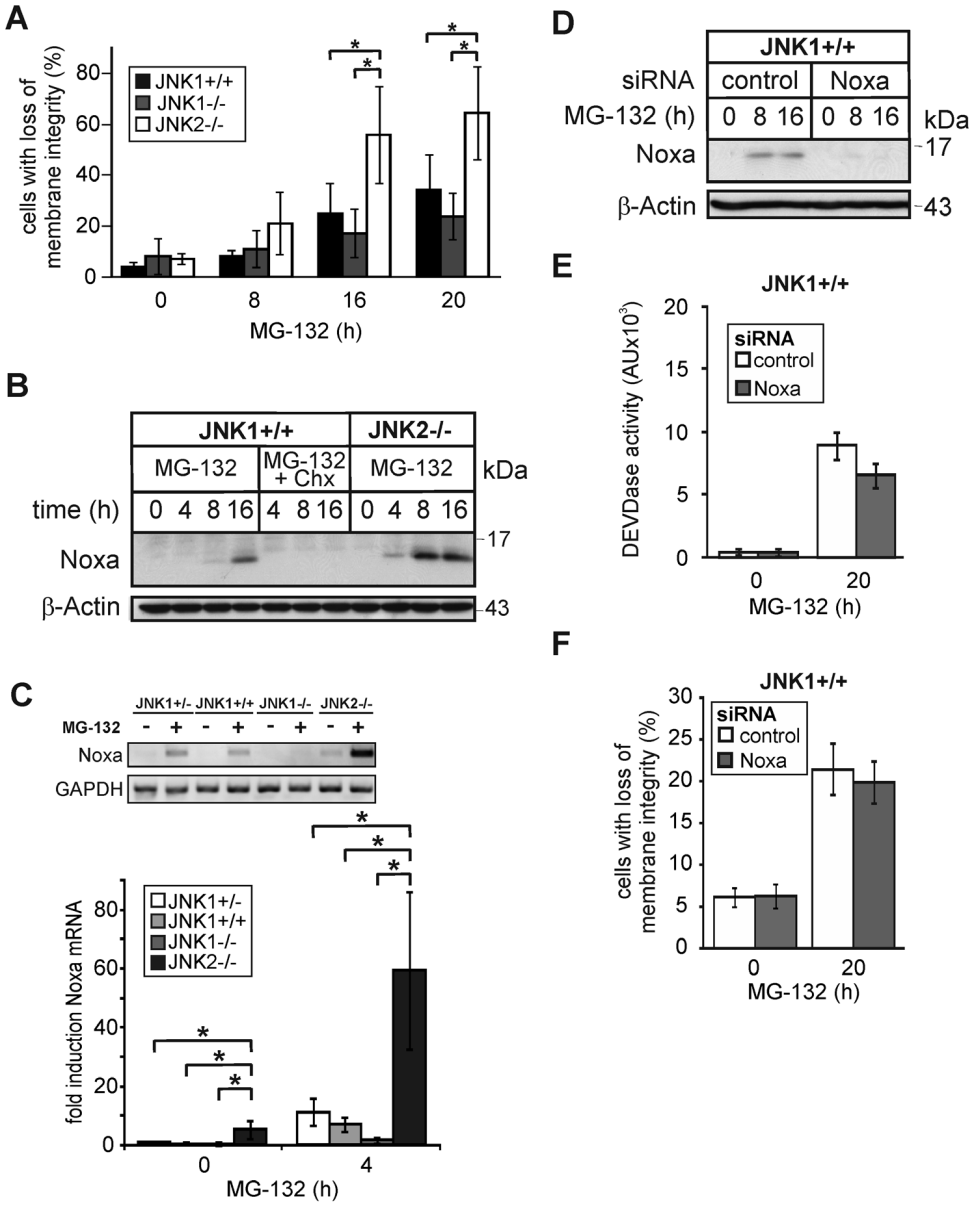
Influence of MG-132 on apoptosis and Noxa expression in JNK1+/+ cells. (**A**) Cytometric determination of cell death (propidium iodide uptake) of JNK1+/+, JNK1−/− and JNK2−/− cells treated with MG-132 as indicated. Values represent the mean of three independent experiments +/− SD. (**B**) Western blots for the status of Noxa and β-actin in JNK1+/+ and JNK2−/− cells treated as indicated. (**C**) Semi-quantitative PCR for the determination of the MG-132-induced expression levels of Noxa mRNA in the indicated MEF lines normalized to GAPDH mRNA. Data are the mean of three independent experiments +/− SD. The inlet shows one representative agarose gel. (**D-F**) Knockdown of Noxa does not protect JNK1+/+ cells from MG-132-induced apoptosis. (**D**) Western blots showing the status of Noxa and β-actin in JNK1+/+ cells that were either left untreated or exposed for the indicated times to MG-132 72 hours post transfection with control or Noxa siRNAs. One representative experiment out of three is shown. (**E**, **F**) Fluorometric and cytometric determination of caspase-3 (DEVDase) activities and cell death (propidium iodide uptake), respectively, in JNK1+/+ cells treated as described in **D**. Values are the mean of three independent experiments +/− SD. For panels A (ANOVA) and C (ANOVA on ranks) * p<0.05 according to the Student-Newman-Keuls-Method in a time-matched comparison as indicated.

Although our data so far indicate that PI-induced apoptosis requires JNK1-dependent Noxa upregulation, they might additionally be interpreted that JNK2 functions in an inhibitory manner. In an attempt to clarify this issue, we examined PI-induced Noxa induction and apoptosis also in JNK1+/+ cells. Remarkably, these cells were clearly less sensitive than similar treated JNK2−/− cells (Fig. 4A). Similar to JNK1+/− cells, this moderate MG-132 sensitivity correlated well not only with a relative weak activation of caspase-3 (data not shown), but also with an intermediate induction of Noxa protein and mRNA when compared to JNK1−/− and JNK2−/− cells (Figs. 4B, 4C). Consistent with our previous data (Fig. 2C), Noxa mRNA induction was most pronounced in JNK2−/− cells, but only barely detectable in JNK1−/− cells (Fig. 4C). In agreement with these findings, knockdown of Noxa had only a marginal effect on MG-132-induced caspase-3 activation and apoptosis in JNK1+/+ cells (Figs. 4D-F).

**Figure 5:**
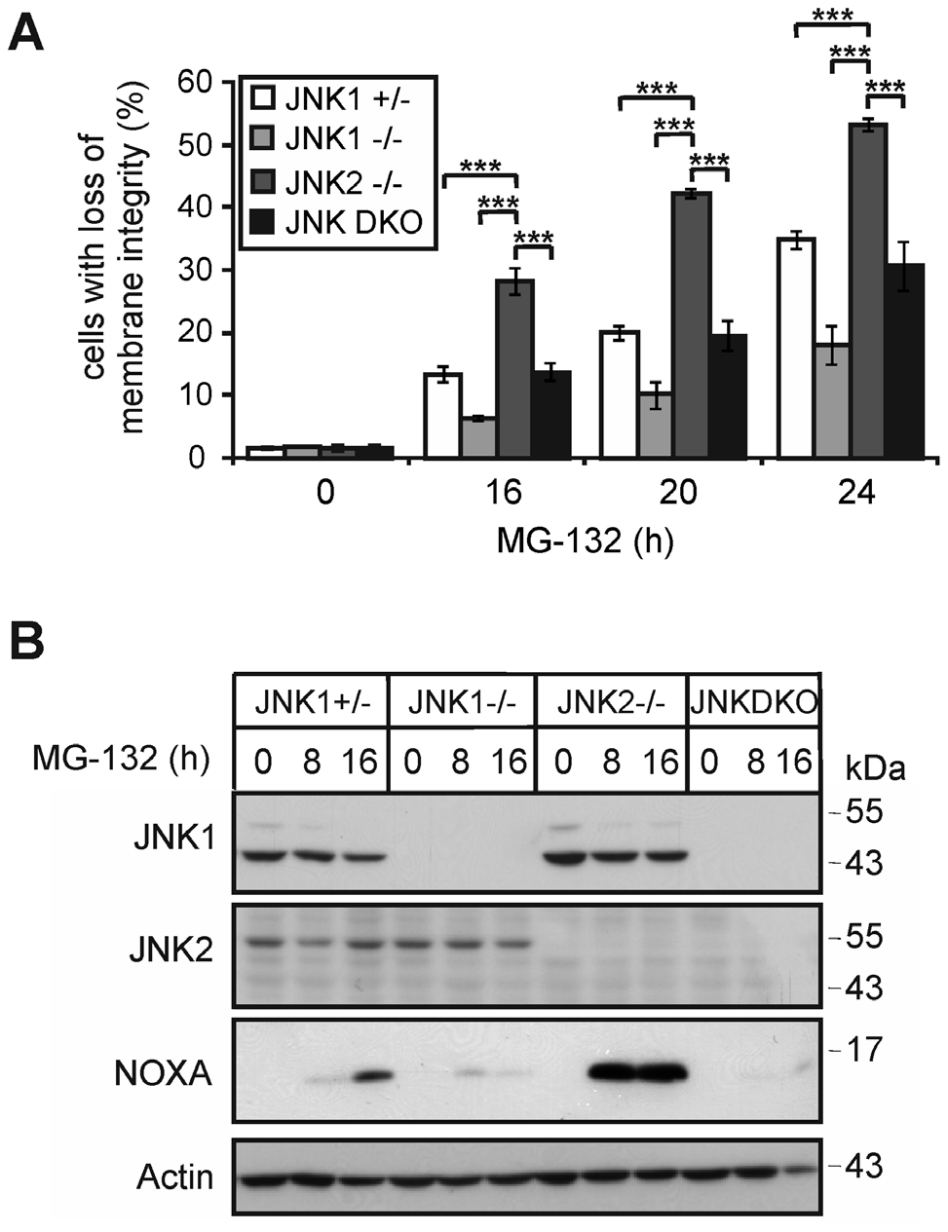
JNK1/2-dependent cell death induction and Noxa expression following MG-132 exposure. (**A**) Cytometric determination of cell death (propidium iodide uptake) of the indicated MEF lines treated with MG-132 as indicated. Values represent the mean of three independent experiments +/− SD. *** p<0.005 according to ANOVA Tukeýs test in a time-matched comparison as indicated. (**B**) Western blots for JNK1, JNK2, Noxa and β-actin in the indicated cell lines treated with MG-132 as indicated. One representative experiment out of three is shown.

To unambigously clarify whether the two JNK isoforms exert oppositional roles in PI-induced apoptosis, we finally exposed cells completely lacking both JNK1 and JNK2 (JNK-DKO) to MG-132. Although JNK-DKO cells exhibited together with JNK1+/− cells an intermediate death response when compared to JNK1−/− and JNK2−/− cells, their PI-induced Noxa protein levels were almost undetectable and thus similar to those observed in JNK1−/− cells (Fig. 5). As Noxa levels increased slightly and even more dramatically in JNK1+/− and JNK2−/− cells, respectively, these data not only support our conclusion that JNK1 is required for PI-induced Noxa expression and apoptosis, but, in addition, provide strong evidence that JNK2 counteracts these responses.

### Knockdown of c-Myc impairs PI-induced apoptosis of JNK2−/− cells without affecting expression of Noxa

**Figure 6:**
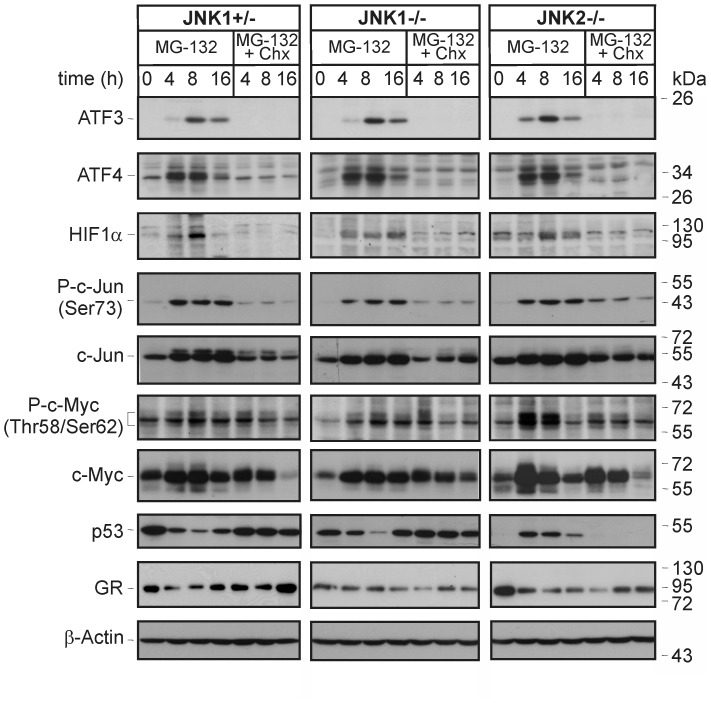
Influence of MG-132 on expression of transcription factors known to be involved in Noxa regulation. Western blot analyses for the indicated transcription factors and β-actin in the three MEF lines treated as indicated. One representative experiment out of three is shown.

Having demonstrated that the JNK1-dependent transcriptional upregulation of Noxa is crucial for PI-induced apoptosis of JNK2−/− MEFs, we aimed to identify the transcription factor responsible for this event. Searching the Genomatix software suite (www.genomatix.de), 85 putative candidates were found to associate with the Noxa gene *PMAIP1*. From these, we closely examined the role of several transcription factors including ATF3, ATF4, c-Jun, c-Myc, HIF1α, p53, and the glucocorticoid receptor (GR), as most of them were not only shown to transcriptionally regulate Noxa expression [17], [27]–[31], and known to be involved in JNK signaling [32]–[37], but also because they represent prominent targets of the proteasome [38]-[44]. In addition, several of these transcription factors were found upregulated in various screens for modulators of cell death induced by PIs [45]–[47]. In agreement with these data we observed that MG-132 induced in all three MEF lines an upregulation of ATF3, ATF4, c-Jun, c-Myc and HIF1α that was abrogated in the presence of cycloheximide (Fig. 6). For unknown reasons, expression of the GR declined under these conditions. Expression of p53, on the other hand, increased only in MG-132-treated JNK2−/− cells and declined in similar treated JNK1+/− and JNK1−/− cells, suggesting that the latter two cell lines most likely harbor a mutant p53 protein. Interestingly, whereas c-Jun was phosphorylated upon MG-132 exposure in all three MEF lines almost to a similar extent, phosphorylation of c-Myc was most prominent in JNK2−/− cells. Together, these data point to c-Myc and p53 as the most likely candidates for the observed JNK1-dependent upregulation of Noxa in response to PIs.

**Figure 7:**
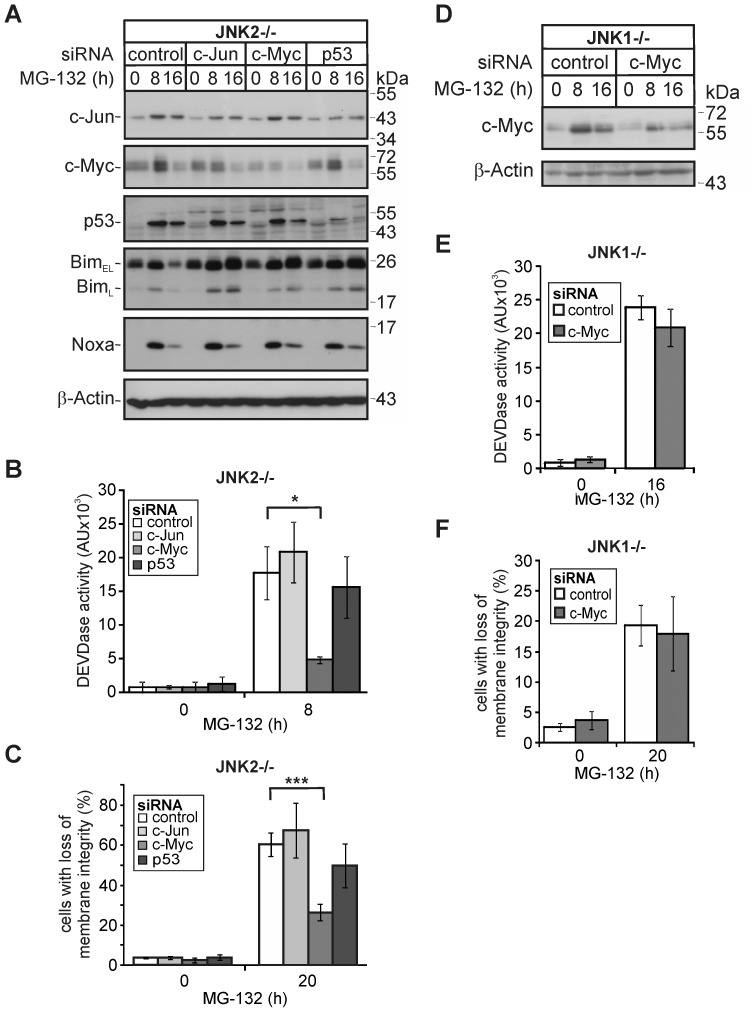
Knockdown of c-Myc protects JNK2−/−, but not JNK1−/− cells from MG-132-induced apoptosis without affecting expression of Noxa and Bim. (A, D) Western blots showing the status of the indicated proteins in JNK2−/− and JNK1−/− cells that were either left untreated or exposed for the indicated times to MG-132 72 hours post transfection with the indicated siRNAs. One representative experiment out of four and three is shown, respectively. (B, E) Fluorometric determination of caspase-3 (DEVDase) activities in JNK2−/− and JNK1−/− cells treated as described in A and D, respectively. Please note that DEVDase activities of JNK2−/− and JNK1−/− cells were analyzed at different time points (8 h and 16 h) according to their sensitivities. Values are the mean of three independent experiments +/− SD, respectively. (C, F) Cytometric determination of cell death (propidium iodide uptake) in JNK2−/− and JNK1−/− cells treated as described in A and D. Values are the mean of three independent experiments +/− SD. * p<0.05 according to ANOVA on ranks Student-Newman-Keuls-Method (B) or *** p<0.003 according to ANOVA Tukey′s test (C) in an all pairwise multiple comparison as indicated.

With the exception of c-Myc, however, none of these transcription factors appear to be involved in MG-132-induced apoptosis of JNK2−/− cells, as their siRNA-mediated knockdown affected neither the MG-132-induced expression of Noxa or Bim, activation of caspase-3 or apoptosis (Figs. 7, S3, S4). Solely the depletion of c-Myc resulted in a strong inhibition of MG-132-induced caspase-3 activation (∼72%) and apoptosis (∼56%) in JNK2−/− cells (Figs. 7B-C). In contrast, the delayed JNK1-independent apoptosis pathway induced by MG-132 in JNK1−/− cells was neither compromised by the knockdown of c-Myc or Noxa (Figs. 3D-F; Figs. 7D-F), ruling out off target effects of these siRNAs and suggesting that both proteins are components of a JNK1-dependent PI-induced apoptosis pathway. Despite these observations, however, knockdown of c-Myc in MG-132-treated JNK2−/− cells did not yield in the expected down regulation of Noxa or Bim (Fig. 7A). Together, these results provide strong evidence that none of the herein examined transcription factors including c-Myc is required for the upregulation of Noxa following PI exposure.

## Discussion

Inhibition of the proteasome either on its own or in combination with other apoptotic stimuli is a powerful means to specifically eradicate tumor cells, but the underlying molecular pathways are only incompletely deciphered [1]. JNKs and the BH3-only protein Noxa were reproducibly demonstrated in many diverse systems to be essential constituents of this process as inhibition of their activity and/or expression substantially protected cells from PI-induced apoptosis [11]–[13], [20], [21]. However, these two pathways have never been connected before and the contributions (if any) of individual JNK isoforms to PI-mediated induction of Noxa and apoptosis were completely unknown.

Using various JNK1- and/or JNK2-deficient fibroblasts, we demonstrate here for the first time that JNK1 is required for PI-induced Noxa expression and subsequent rapid induction of apoptosis. This is evidenced by our findings that Noxa mRNA and protein levels were almost undetectable in PI-treated JNK1−/− cells, but were induced in similar treated cells expressing at least one allele of JNK1. Intriguingly, as the JNK1-dependent Noxa expression was most dramatically induced in the absence of JNK2 (∼ 40fold in JNK2−/− cells compared to ∼ 6–8fold in either JNK1+/− or JNK1+/+ cells), these findings additionally imply a negative regulatory role for JNK2 in this event. Finally, both JNK1-deficient MEF lines (JNK-DKO and JNK1−/−) failed to upregulate Noxa following PI exposure, further suggesting that withdrawing the inhibitory effect of JNK2 is by itself not sufficient to launch expression of Noxa in the concurrent absence of JNK1. Consistently, JNK2−/− cells succumbed rapidly to apoptosis following PI treatment, whereas several hallmarks of apoptosis appeared only delayed and severely reduced in their intensities in similar treated cells expressing JNK2. Based on these results and our finding that knockdown of Noxa severely compromised PI-induced apoptosis of JNK2−/− cells, we postulate that the opposing functions of JNK1 and JNK2 in the regulation of Noxa expression represent crucial events in this cell death pathway.

Although so far, JNK1 and JNK2 have mostly been considered to exert overlapping or even redundant functions, a few studies have recently described opposing effects for these kinases that are in line with our observations. Particularly with regard to their involvement in numerous cell death systems, JNK1 and JNK2 were shown to differentially regulate expression and/or function of their targets p53, c-jun and Elk-1 resulting in an oppositional modulation of stress- and basal (non-stress)-induced apoptosis and RNA polymerase III-dependent transcription [48]–[50]. The mechanisms involved, however, were not well characterized leaving the question open whether JNK1 and JNK2 mediate these oppositional effects directly by phosphorylating diverse sites in these targets, or indirectly by modulating additional components that function in an inhibitory or stimulatory manner. In any case, individual JNKs may harbour intrinsic target site specificities or their activities may be regulated differentially by other factors. With regard to the latter scenario we have recently shown that the cyclin-dependent kinase (CDK) inhibitor p21 can differentially modulate the activities of certain kinases including those of several JNK1/2 isoforms in a remarkable substrate-dependent manner [51]. Whether this or other mechanisms are involved in the oppositional regulation of Noxa expression by JNK1 and JNK2 remains to be elucidated.

Remarkably, despite the fact that all three JNK isoforms are able to promote or induce apoptosis, similar to the findings in our study, it was particularly JNK1 that has been implicated in different apoptosis pathways including those instigated by TNFα, UV irradiation and nitric oxide [25], [26], [52]. Employing JNK-deficient cells it was for instance shown that JNK1 promotes TNFα killing by phosphorylation and activation of the ubiquitin ligase Itch that mediates the proteasome-dependent degradation of the caspase-8 inhibitor FLIP [53]. During UV- and nitric oxide-induced apoptosis on the other hand, it was demonstrated that the JNK1-dependent phosphorylation of the anti-apoptotic myeloid cell leukemia-1 (Mcl-1) protein results in its proteasomal degradation [54], [55]. Interestingly, as Mcl-1 is the major counterpart of Noxa and its loss is a critical event that leads to activation of Bax and Bak [56], [57], its JNK1-dependent elimination shifts the delicate Mcl-1-Noxa balance toward apoptosis induction as observed in many systems including those instigated by UV irradiation [58], [59]. Although for obvious reasons proteasomal degradation of Mcl-1 does not contribute to PI-induced apoptosis, the herein described massive upregulation of Noxa (40-fold) is surely able to efficiently bypass this shortcoming by directly counteracting anti-apoptotic Bcl-2 proteins including Mcl-1. This view is supported by our finding that MG-132 induced similar Mcl-1 levels independently of JNK1/2, further emphasizing that the JNK1/2-dependent regulation of Noxa represents the most crucial event in the herein uncovered PI-induced apoptosis pathway. Furthermore, once activated, the mitochondrial death cascade then causes elimination of Mcl-1, as this anti-apoptotic Bcl-2 protein was shown to become proteolytically inactivated during PI-induced apoptosis in a caspase-3-dependent manner [60], [61]. Intriguingly, JNK1−/− mice are highly susceptible to DMBA/PMA-induced skin tumor formation when compared to similar treated wild type mice [62]. As both JNKs and the human Noxa orthologue PMAIP1 (PMA-inducible protein 1) can be strongly activated by phorbol esters, it is tempting to speculate that this tumor suppressive function of JNK1 depends on the induction of Noxa. As, however, JNKs including JNK1 are able to exert also oncogenic functions, that were particularly evident in the development of human hepatocellular carcinoma [63], it is highly likely that JNK1 mediates its versatile functions strictly in a cell type-dependent manner.

Besides being reported as a PMA-inducible protein, Noxa was originally identified as a p53-induced stress response gene, but is now known to be regulated by an array of different transcription factors independently of p53 [17], [64]. However, although several transcription factors known to participate in JNK signaling and diverse apoptosis pathways, and that were even shown to constitute prominent targets of the proteasome, none of those examined here including c-Jun, c-Myc, Hif1α, ATF3, ATF4 and GR appear to be involved in the herein observed JNK1/2-dependent opposite regulation of Noxa. A participation of p53 cannot be completely ruled out, as a few MEF lines studied here may harbor p53 mutations. Perhaps most unexpected was our finding that even the knockdown of c-Myc, a transcription factor that was recently demonstrated to transcriptionally upregulate Noxa during bortezomib-induced apoptosis [29], did not affect Noxa expression despite the observed protection of JNK2−/− cells from PI-induced apoptosis. Particularly with regard to the fact that JNKs phosphorylate and thereby regulate the apoptotic function of c-Myc [34], its previous identification as a potent Noxa inducer upon PI treatment provided strong evidence for the existence of a JNK-Myc-Noxa axis, at least in melanoma cells [29]. As the thereby identified c-Myc binding site in the Noxa promoter is conserved among human, mouse and rat, it is presently unknown why c-Myc induces expression of Noxa only in human melanoma cells, but not in the MEF lines studied here.

Also cells lacking JNK1 eventually succumb to apoptosis in almost the complete absence of Noxa, albeit in a delayed manner. Together with the observation that knockdown of Noxa had no effect on the delayed JNK1/2-independent cell death pathway occurring most likely in all here examined MEF lines following their exposure to PIs, our results strongly support the existence of alternative PI-induced pathways that kill cells independently of JNKs and Noxa. The BH3-only protein Bim might be part of such a pathway, as it was found by us and others upregulated in response to PIs in a JNK-independent manner, and its knockdown partially protected JNK2−/− cells from PI-induced apoptosis [9]. Furthermore, the PI-mediated upregulation of Bim was, unlike the induction of Noxa, not entirely blocked in the presence of cycloheximide. This suggests that the increase in Bim protein expression is probably a direct effect of proteasome inhibition that prevents degradation of this pro-apoptotic BH3-only molecule [9]. Thus, Bim most likely represents an alternative route to cell death in cases in which PIs are unable to mediate the JNK1-dependent upregulation of Noxa.

In summary, we have shown here that a rapid PI-induced apoptosis pathway critically depends on the induction of Noxa that is controlled by JNK1 and JNK2 in an opposing manner. Although we were unable so far to identiy the transcription factor(s) involved, our results might help to further improve future anticancer strategies that are based on proteasomal inhibitors. Thereby, one should keep in mind that our observations are solely based on the use of immortalized MEFs. To exclude possible phenotypical changes acquired during their immortalization, it will be necessary to confirm these findigs using primary MEFs or lymphocytes from JNK1/2 knockout mice.

## Materials and Methods

### Cell lines, reagents and antibodies

Immortalized MEFs (JNK1+/+, JNK1+/−, JNK1−/−, JNK2−/−, JNK-DKO) [26] were maintained in high glucose DMEM supplemented with 10% heat-inactivated fetal calf serum, 10 mM glutamine, 100 U/ml penicillin and 0.1 mg/ml streptomycin (PAA Laboratories, Linz, Austria). The pan-caspase inhibitory peptide Q-VD-OPh (Q-Val-Asp-CH_2_-O-Ph) was from MP Biomedicals (Irvine, CA). The fluorogenic caspase-3 substrate DEVD-AMC (N-acetyl-Asp-Glu-Val-Asp-aminomethylcoumarin) and the calpain inhibitors I (ALLN) and II (ALLM) were from Biomol (Hamburg, Germany), whereas the proteasomal inhibitors MG-132, clasto-lactacystin β-lactone (CLC) and bortezomib were purchased from Enzo Life Sciences (Lörrach, Germany), Boston Biochem (Wiesbaden, Germany), and from LC Laboratories (Woburn, MA, USA), respectively. The monoclonal antibodies against Bim, c-Jun, and caspase-3 as well as the polyclonal antibodies recognizing JNK2, c-Myc, or phospho-specific sites in c-Jun and c-Myc were from Cell Signaling Technology (Danvers, MA, USA). From BD Biosciences (Heidelberg, Germany) we purchased the monoclonal and polyclonal antibodies to JNK1 and Bcl-xL, respectively, whereas polyclonal Bax and Bak antibodies were from Millipore (Billerica, MA, USA). The polyclonal rabbit antibodies against Mcl-1 and HIF1α were from Rockland Immunochemicals (Gilbertsville, PA, USA) and Novus Biologicals (Littleton, CO, USA, respectively. The monoclonal β-actin and p53 antibodies were from Sigma-Aldrich (Deisenhofen, Germany) and Merck Chemicals Ltd. (Nottingham, UK), respectively. From Santa Cruz Biotechnology (Santa Cruz, CA, USA) we obtained the polyclonal Noxa, ATF3, ATF4 and glucocorticoid receptor antibodies as well as the monoclonal Bcl-2 antibody. Actinomycin D (ActD), cycloheximide (Chx), propidium iodide, and the protease inhibitors PMSF, aprotinin, leupeptin and pepstatin were from Sigma (Deisenhofen, Germany). Peroxidase-labeled secondary antibodies were from Promega GmbH (Mannheim, Germany).

### Treatment of cells and measurement of cell death

Cells were usually treated with 10 µM MG-132 in the absence or presence of cycloheximide (Chx; 10 µg/ml), or the pan caspase inhibitor Q-VD-OPh (10 µM). Treatment with other proteasomal inhibitors is specified. Cell death was analysed cytometrically either by the uptake of propidium iodide (2 µg/ml in phosphate-buffered saline) to determine the percentage of cells with a loss of membrane integrity, or by quantifying the proportion of nuclei containing hypodiploid DNA by lysing cells in a hypotonic buffer containing 0.1% sodium citrate, 0.1% Triton X-100, and 50 µg/ml propidium iodide. The mitochondrial transmembrane potential (ΔΨ_m_) was analyzed by incubating cells with 25 nmol/L of the ΔΨ_m_-specific stain TMRE (Molecular Probes, Eugene, OR, USA) for 30 minutes. All flow cytometric analyses were performed on a FACSCalibur (Becton Dickinson, Heidelberg, Germany) with the CellQuest Pro analysis software. For each determination, a minimum of 10,000 cells was analysed.

### Preparation of cell extracts and Western blotting

Total cell extracts were prepared in lysis buffer containing 1% NP-40, 50 mM Tris-HCl (pH 7.4), 150 mM NaCl, 1 mM DTT, and protease and phosphatase inhibitors. Protein concentrations were determined with the BioRad protein assay. Subsequently, proteins were separated on SDS-polyacrylamide gels and electroblotted to polyvinylidene difluoride membranes (Millipore, Schwalbach, Germany). Following antibody incubation, the proteins were visualized by enhanced chemiluminescent staining using ECL reagents (Amersham Biosciences).

### Fluorometric determination of caspase activity

Caspase-3 activities were assessed by using 50 µg of the cell extracts that were directly dissolved in 200 µl substrate buffer (50 mM HEPES pH 7.3, 100 mM NaCl, 10% sucrose, 0.1% CHAPS, 10 mM DTT) supplemented with 50 µM DEVD-AMC (Biomol). The reaction was incubated at 37°C for 3 h and the release of fluorogenic AMC was measured at an excitation wavelength of 346 nm and an emission wavelength of 442 nm using an measured in a Infinite M200 microplate reader (Tecan, Langenfeld, Germany). The detected fluorometric signal that directly correlates to the caspase activity in the cell extracts is expressed in arbitrary units (AU).

### Determination of Noxa and Bim mRNA expression by semi-quantitative PCR analysis

Cells were exposed to MG-132 for 4 hours and total mRNA was isolated with the RNeasy mini kit (Qiagen, Hilden, Germany) according to the instructions of the manufacturer. 2 µg of the isolated RNA was transcribed into cDNA using random hexamers (Applied Biosystems, Darmstadt, Germany) as primers and MMLV reverse transcriptase (Invitrogen, Karlsruhe, Germany). 100 ng of the resulting cDNA was subjected to semi-quantitative PCR for 25 cycles using Taq Polymerase (5 Prime, Hamburg, Germany) and specific exon-spanning primers for the mRNAs of Noxa and Bim as well as for GAPDH as an endogenous control. One half of each PCR reaction was separated on an ethidium bromide-stained agarose gel and the corresponding bands were densitometrically analyzed using a CCD camera (LAS-4000) together with the Multi Gauge software, both from Fujifilm Life Science (Düsseldorf, Germany). The signal of each band was normalized to the corresponding GAPDH band.

### Transfection of siRNAs

ON-TARGETplus SMARTpool and non-targeting control siRNAs were purchased from Dharmacon RNA technologies (Lafayette, CO, USA), and the knockdown was carried out according to the manufacturer’s instructions. Fourty-eight to seventy-two hours after transfection, cells were divided equally to receive either no treatment or exposure to proteasomal inhibitors. Cells were harvested at the indicated time points and directly analysed by Western blotting, by the fluorometric caspase substrate assay, or cytometrically for a successful knockdown of the target protein, DEVDase activity and cell death, respectively.

### Statistical Analyses

Data, expressed as means ± SD, were analyzed statistically using SigmaPlot 12.3. Shapiro-Wilk test was performed for normality testing. Equal variance was checked by Spearman rank correlation. One-way analysis of variance (ANOVA) as a parametric test or Kruskal-Wallis one-way analysis of variance on ranks as a non-parametric test for comparison of differences between measurements was used. p values of less than 0.05 were considered statistically significant. For post-hoc comparison Dunńs, Tukey or Student-Newman-Keuls test were employed.

## Supporting Information

Figure S1 (**A** and **B**) Rapid induction of apoptosis by MG-132 only in JNK2−/− cells. Flow cytometric (propidium iodide uptake) (**A**) and fluorometric (caspase-3-like DEVDase activity) (**B**) cell death determination of JNK1+/−, JNK1−/− and JNK2−/− MEFs that were exposed to MG-132 for the indicated times. Values represent the mean of three independent experiments +/− SD. (**C**) Proteasomal inhibitors preferentially induce cell death in JNK2−/− MEFs. Flow cytometric determination of cell death (propidium iodide uptake) in JNK1+/−, JNK1−/− and JNK2−/− cells that were either left untreated or exposed for 24 hours to the indicated compounds. Values are the mean of three independent experiments +/− SD. For all panels, * p<0.05; ** p<0.01; *** p<0.005 according to ANOVA or ANOVA on ranks Tukeýs test in a time- (A and B) and dose-matched (C) comparison of JNK2−/− vs. JNK1+/− and JNK1−/−, respectively. Please note that panels A to C are alternative presentations of some of the data of Fig. 1A, 1B and Suppl. Fig. 2, respectively.
(TIF)

Figure S2 Various proteasomal inhibitors including bortezomib, ALLN and clasto-lactocystine β-lactone (CLC) rapidly induce cell death preferentially in JNK2−/− MEFs. Flow cytometric determination of cell death (propidium iodide uptake) in JNK1+/−, JNK1−/− and JNK2−/− cells that were either left untreated or exposed for 24 hours to the indicated compounds in the absence and presence of cycloheximide. Values are the mean of three independent experiments +/− SD. For all panels, * p<0.05; ** p<0.01; *** p<0.005 according to ANOVA Tukeýs test in a dose-matched comparison vs. Chx treatment.
(TIF)

Figure S3 Knockdown of ATF3 and ATF4 has no effect on apoptosis, caspase-3 activation or expression of Noxa and Bim in MG-132-treated JNK2−/− cells. (**A**) Western blots showing the status of the indicated proteins in JNK2−/− cells that were either left untreated or exposed for the indicated times to MG-132 72 hours post transfection with control, ATF3 or ATF4 siRNAs. One representative experiment out of three is shown. (**B** and **C**) Fluorometric and flow cytometric determination of caspase-3 (DEVDase) activities and cell death (propidium iodide uptake), respectively, in JNK2−/− cells that were treated as described in **A**. Values are the mean of three independent experiments +/− SD.
(TIF)

Figure S4 Knockdown of Hif1α and the glucocorticoid receptor (GR) has no effect on apoptosis, caspase-3 activation or expression of Noxa and Bim in MG-132-treated JNK2−/− cells. (**A** and **D**) Western blots showing the status of the indicated proteins in JNK2−/− cells that were either left untreated or exposed for the indicated times to MG-132 72 hours post transfection with control, Hif1α or GR siRNAs. One representative experiment out of three is shown. (**B, C, E, F**) Fluorometric and flow cytometric determination of caspase-3 (DEVDase) activities and cell death (propidium iodide uptake), respectively, in JNK2−/− cells that were treated as described in **A** and **D**. Values are the mean of three independent experiments +/− SD.
(TIF)
