# Supplementary figures and images for: Oppositional Regulation of Noxa by JNK1 and JNK2 during Apoptosis Induced by Proteasomal Inhibitors
Source: PLoS One. 2013 Apr 11;8(4):e61438. doi: 10.1371/journal.pone.0061438 (PMC3623862; doi:10.1371/journal.pone.0061438)

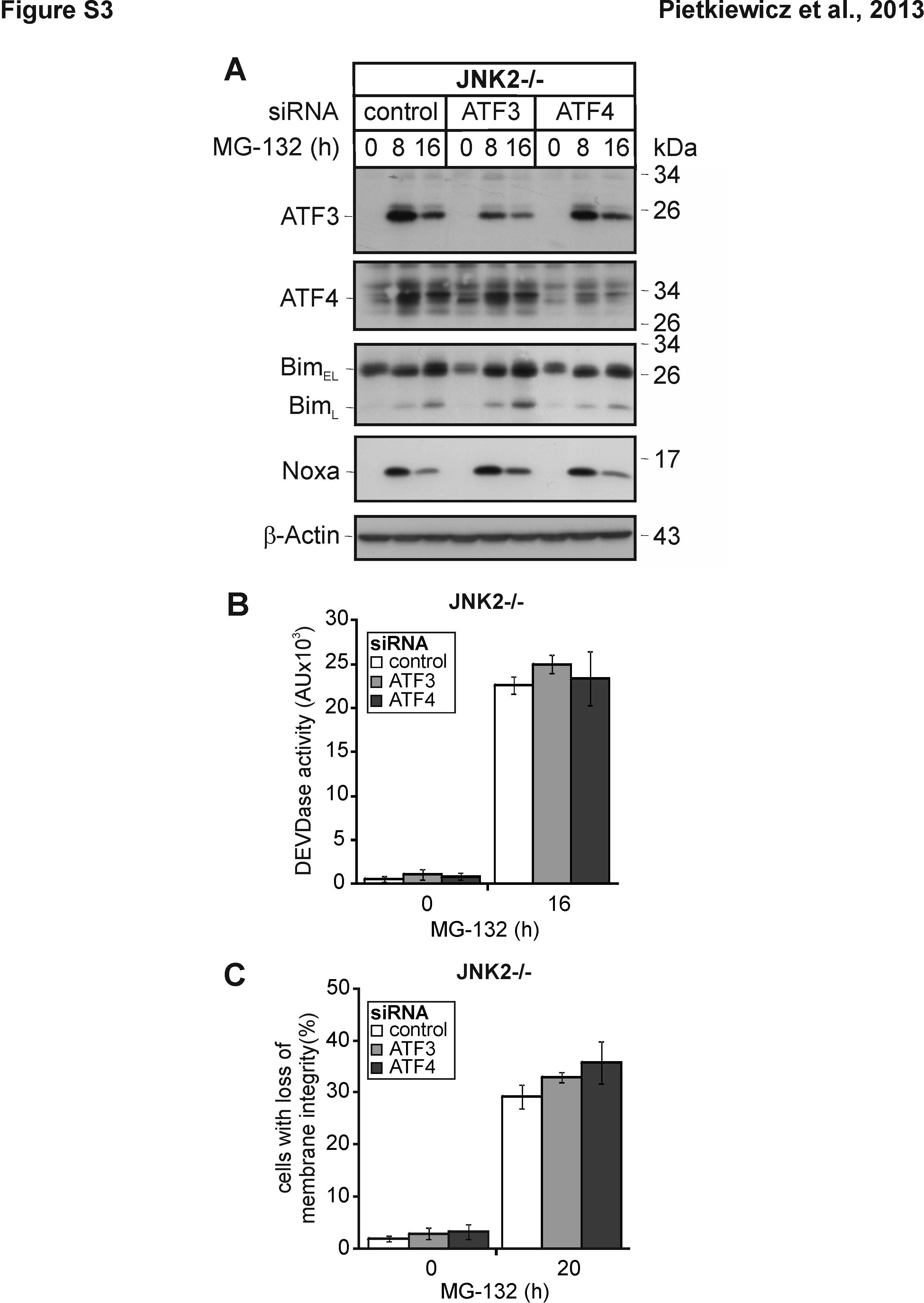

Supplement: Figure S3 — Knockdown of ATF3 and ATF4 has no effect on apoptosis, caspase-3 activation or expression of Noxa and Bim in MG-132-treated JNK2−/− cells. (A) Western blots showing the status of the indicated proteins in JNK2−/− cells that were either left untreated or exposed for the indicated times to MG-132 72 hours post transfection with control, ATF3 or ATF4 siRNAs. One representative experiment out of three is shown. (B and C) Fluorometric and flow cytometric determination of caspase-3 (DEVDase) activities and cell death (propidium iodide uptake), respectively, in JNK2−/− cells that were treated as described in A. Values are the mean of three independent experiments +/− SD. (TIF) [file pone.0061438.s003.tif]

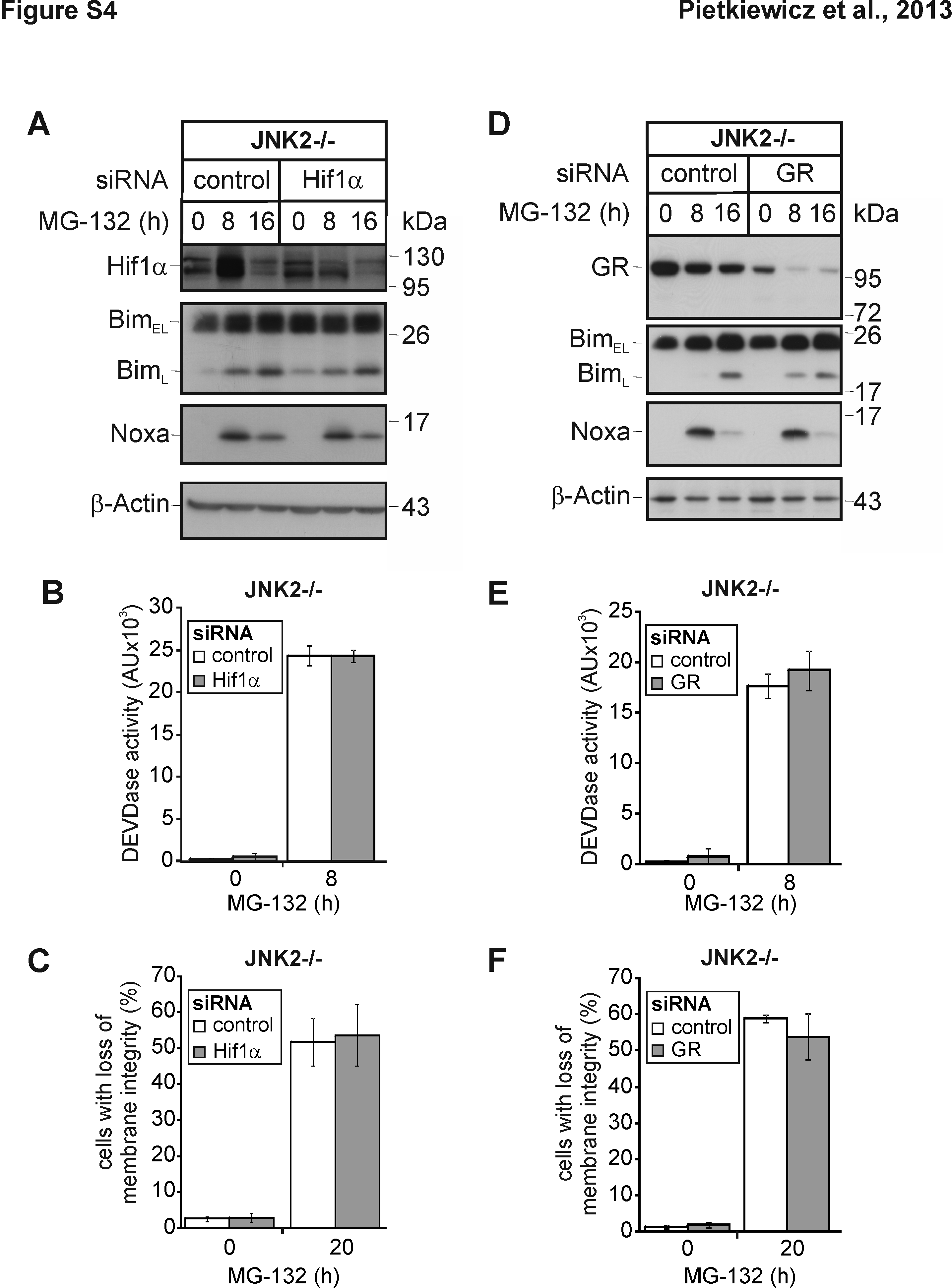

Supplement: Figure S4 — Knockdown of Hif1α and the glucocorticoid receptor (GR) has no effect on apoptosis, caspase-3 activation or expression of Noxa and Bim in MG-132-treated JNK2−/− cells. (A and D) Western blots showing the status of the indicated proteins in JNK2−/− cells that were either left untreated or exposed for the indicated times to MG-132 72 hours post transfection with control, Hif1α or GR siRNAs. One representative experiment out of three is shown. (B, C, E, F) Fluorometric and flow cytometric determination of caspase-3 (DEVDase) activities and cell death (propidium iodide uptake), respectively, in JNK2−/− cells that were treated as described in A and D. Values are the mean of three independent experiments +/− SD. (TIF) [file pone.0061438.s004.tif]
